# Amino acid modified OCMC-g-Suc-*β*-CD nanohydrogels carrying lapatinib and ginsenoside Rg1 exhibit high anticancer activity in a zebrafish model

**DOI:** 10.3389/fphar.2023.1149191

**Published:** 2023-05-11

**Authors:** Li Cui, Xiaolan Liu, Rongjun Yan, Qixu Chen, Lizhen Wang, Shah Nawaz, Dawei Qin, Daijie Wang

**Affiliations:** ^1^ School of Chemistry and Chemical Engineering, Qilu University of Technology (Shandong Academy of Sciences), Jinan, China; ^2^ Shandong Analysis and Test Center, Qilu University of Technology (Shandong Academy of Sciences), Jinan, China; ^3^ Jinan Authority Hospital, Jinan, China; ^4^ Jinan International Travel Healthcare Center, Jinan, China; ^5^ Biological Engineering Technology Innovation Center of Shandong Province, Heze Branch of Qilu University of Technology (Shandong Academy of Sciences), Heze, China; ^6^ Biology Institute, Qilu University of Technology (Shandong Academy of Sciences), Jinan, China; ^7^ Department of Chemistry, Karakoram International University, Gilgit, Pakistan

**Keywords:** OCMC-g-*β*-CD nanohydrogels, lapatinib, ginsenoside Rg1, anticancer activity, zebrafish

## Abstract

Nanohydrogels show great potential as efficient drug carriers due to their biocompatibility, low toxicity, and high water absorbability. In this paper, we prepared two *O*-carboxymethylated chitosan (OCMC)-based polymers functionalized with β-cyclodextrin (β-CD) and amino acid. The structures of the polymers were characterized by Fourier Transform Infrared (FTIR) Spectroscopy. Morphological study was carried out on a Transmission Electron Microscope (TEM), and the results indicated that the two polymers had irregular spheroidal structure with some pores distributed on their surface. The average particle diameter was below 500 nm, and the zeta potential was above +30 mV. The two polymers were further used for preparing nanohydrogels loaded with anticancer drugs lapatinib and ginsenoside Rg1, and the resulting nanohydrogels showed high drug loading efficiency and pH-sensitive (pH = 4.5) drug release behavior. *In vitro* cytotoxicity investigation revealed that the nanohydrogels exhibited high cytotoxicity against lung cancer (A549) cells. *In vivo* anticancer investigation was performed in a transgenic *Tg*(*fabp10:rtTA2s-M2; TRE2:EGFP-kras* ^*V12*^) zebrafish model. The results showed that the synthesized nanohydrogels significantly inhibited the expression of *EGFP-kras* ^*v12*^ oncogene in zebrafish liver, and the *L*-arginine modified OCMC-g-Suc-*β*-CD nanohydrogels loading lapatinib and ginsenoside Rg1 showed the best results.

## Introduction

Nanohydrogels exhibit great potential for developing drug delivery systems due to their promising biocompatibility, low toxicity, and good water absorbability (Bourbon et al., 2016; Swain and Prusty, 2018). Nanohydrogels contain a three-dimensional network structure with a large number of hydrophilic groups, such as −OH, NH_2_, and −CO_2_H (Kabanov and Vinogradov, 2009; Lian et al., 2010). The presence of hydrophilic groups endows nanohydrogels with excellent swelling behavior in water. Therefore, the anticancer drugs can be entrapped and stabilized in the three-dimensional network structure of nanohydrogels through hydrogen-bond complexation and van der Waals’ force (Liu et al., 2016; Humenik et al., 2020; Chander and Kulkarni, 2021).

Chitosan (CS) is an amino-rich polysaccharide obtained by deacetylation of chitin presented in cuticles of crustaceans (Bourbon et al., 2018; Mattia et al., 2019). Under acidic condition, CS can form nanohydrogels due to the formation of NH^3+^ groups (Jain et al., 2012; Jiang et al., 2014; Zhang et al., 2018). More importantly, the primary amino groups in CS can react with various functional groups to produce many CS derivatives, which bear complex three-dimensional network structures and should be suitable for developing novel drug delivery systems (Izawa et al., 2016; Bi et al., 2020). As a drug carrier, CS exhibits excellent bioadhesive properties due to the strong and attractive intermolecular forces, which can help to penetrate epithelial cell barriers and promote drug transportation (Kas, 1997; Akbari-Alavijeh et al., 2020). Therefore, CS and its derivatives have been widely used for preparing different types of nanohydrogels as novel drug delivery systems in the past several years.

However, using naturally occurring CS as a drug carrier is limited due to the presence of disadvantages including the poor water retention capacity and the low drug loading efficiency of CS nanohydrogels (Kono and Teshirogi, 2015; Du et al., 2019). CS must be dissolved in acidic solutions to form hydrogels, whereas some drugs show poor stability under acidic conditions (Liu et al., 2007; Ganguly et al., 2014). Structural modification of CS is interesting and attractive, as the water solubility of CS can be significantly enhanced by introducing hydrophilic groups (Lin et al., 2005; Wu et al., 2014; Wei et al., 2020). Some acidic functional groups can be introduced to CS, and the resulting polymer can form stable hydrogels under neutral pH conditions due to the coexistence of carboxyl and amino groups (Anitha et al., 2011; Du et al., 2014). Consequently, structural modification can solve the problem of poor drug loading efficiency of CS and avoid the degradation of acid-instability drugs.

**FIGURE 1:**
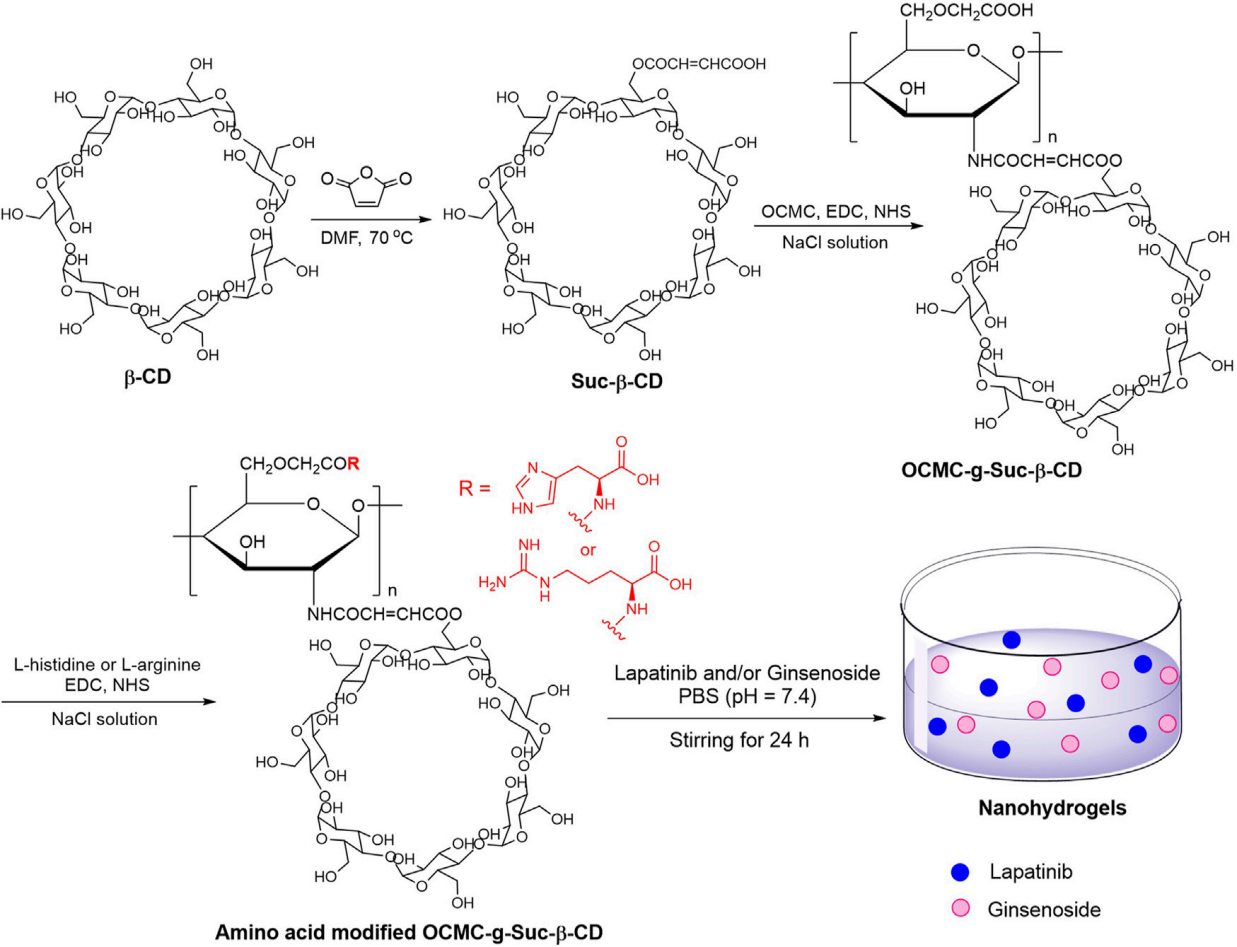
Preparation of amino acid modified OCMC-g-Suc-*β*-CD nanohydrogels carrying anticancer drug.

In this paper, the structure of CS was modified with carboxymethyl groups, to form a water soluble CS derivative carboxymethylated chitosan (OCMC). The amino groups in OCMC was partially modified with *β*-cyclodextrin (*β*-CD), which had a unique hydrophobic cavity that can load many hydrophobic drugs with poor water solubility (Rekharsky and Inoue, 1998; Li et al., 2018). The presence of *β*-CD has been proved to significantly enhanced the drug loading efficiency due to its hydrophobic cavity structure (Liao et al., 2020). In addition, the structure of OCMC was also modified with commercial available amino acid (L-histidine and L-arginine). The two amino acids have been proved to participate in many kinds of interactions between different biomacromolecules in the human body, and therefore have been used as interesting functional groups for improving the biocompatibility of drug carriers (Choi et al., 2012; Hu et al., 2018; Huang et al., 2019; Yogesh et al., 2021). The OCMC polymers modified with *β*-CD and amino acids were further used as drug carriers to load anticancer drugs lapatinib and ginsenoside Rg1 (Figure 1). *In vitro* cytotoxicity study was carried out on an A549 cell line through the well-proved MTT assay, and *in vivo* anticancer study was performed on a transgenic *Tg*(*fabp10:rtTA2s-M2; TRE2:EGFP-kras* ^*V12*^) zebrafish model. The results indicated that the nanohydrogels (**6**) loaded with two anticancer drugs (lapatinib and ginsenoside Rg1) showed high anticancer acitivity in zebrafish model.

## Materials and methods

### Materials and reagents

OCMC (NO. C914893) was obtained from Shanghai Macklin Biochemical Co., Ltd. *β*-CD, lapatinib, *L*-histidine and *L*-arginine were purchased from Shanghai Macklin Biochemical Co., Ltd. *N*, *N*-dimethylformamide (DMF, NO. 227056) was bought from Merck Co., Ltd. 3-(4,5-Dimethylthiazol-2-yl)-2,5-diphenyltetrazolium bromide (MTT, NO. M8180) was bought from Beijing Solarbio Science Technology Co., ltd. FTIR spectra were analyzed using Shimadzu IRPrestige-21 equipment. TEM images were recorded on a Hitachi-7700 transmission electron microscope. Ultrapure water was obtained using a UPT-II-5T water purification system (Ulupure, Chengdu, China). The diameter and zeta potential of the copolymer were measured on a Malvern Zetasizer Nano ZS-90 technique. Cells were contained in DMEM media with 10% FBS and 1% penicillin/streptomycin, and OD value was analyzed on a Microplate Reader (DYNEX, Series1SPA-0093). Ginsenoside Rg1 (NO. PHR2200) was obtained from Merck Co., Ltd. with purity over 98%.

### Preparation of maleic anhydride modified *β*-CD (Suc-*β*-CD)

Suc-β-CD was synthesized according to a published method (Xie et al., 2019). In brief, the solution of *β*-CD (22.80 g) and maleic anhydride (19.61 g) in anhydrous DMF (250 mL) was stirred at 70°C for 12 h. The reaction was then cooled to room temperature and poured into 1,000 mL CH_2_Cl_2_. The resulting mixture was stirred at RT for 20 min and filtered. The white solid was washed with acetone (50 mL × 3) and dried under vacuum to give Suc-*β*-CD.

### Preparation of OCMC-g-Suc-*β*-CD

OCMC-g-Suc-β-CD was synthesized by a published method but with some alterations (Mani and Gong, 2008). To the solution of Suc-*β*-CD (2.46 g) in an aqueous NaCl solution (100 mL, 0.1 mol/L) was added 1-ethyl-3-(3-dimethylaminopropyl)carbodiimide hydrochloride (EDCI, 1.14 g), and the mixture was stirred at RT for 30 min to activate the carboxylic group. Then, *N*-hydroxy succinimide (NHS, 1.15 g) was added and the mixture was stirred for another 30 min, which was followed by addition of OCMC. The mixture was stirred at RT for 12 h and cooled to room temperature. The resulting solution was dialyzed for 72 h using a dialysis bag (3500 MW cutoff), and then lyophilized at −40°C in a vacuum freeze-dryer to provide OCMC-g-Suc-*β*-CD as a white solid.

### Preparation of amino acid modified OCMC-g-Suc-*β*-CD

To the solution of OCMC-g-Suc-*β*-CD (2.00 g) in an aqueous NaCl solution (100 mL, 0.1 mol/L) was added EDCI (1.16 g), and the reaction was allowed to warm to 37°C and stirred at ambient temperature for 30 min. Then, NHS (1.38 g) was added and the mixture was stirred for another 30 min. Thereafter, *L*-histidine (0.93 g) or *L*-arginine (1.04 g) was added and the mixture was stirred at 37°C for 10 h. The reaction was cooled to room temperature and dialyzed for 72 h using a dialysis bag (3,500 MW cutoff). The resulting mixture was lyophilized at −40°C in a vacuum freeze-dryer to provide the amino acid-modified OCMC-g-Suc-*β*-CD (*L*-His-OCMC-g-Suc-*β*-CD, or *L*-Arg-OCMC-g-Suc-*β*-CD).

### Preparation of nanohydrogels **1**-**6**

Preparation of nanohydrogels **1** (*L*-His-OCMC-g-Suc-*β*-CD carrying Lapatinib) and **2** (*L*-His-OCMC-g-Suc-*β*-CD carrying ginsenoside Rg1): The *L*-histidine modified OCMC-g-Suc-*β*-CD (100 mg) was dissolved in a PBS solution (100 mL, 10 mM, pH = 7.4). To the mixture was added anticancer drug Lapatinib (50 mg) or Ginsenoside (50 mg), which was followed by stirring at 25°C for 24 h to form a translucent dispersion.

Nanohydrogels **3** (*L*-Arg-OCMC-g-Suc-*β*-CD carrying lapatinib) and **4** (*L*-Arg-OCMC-g-Suc-*β*-CD carrying ginsenoside Rg1) were synthesized by a same procdure described above using *L*-arginine modified OCMC-g-Suc-*β*-CD as carrier.

Preparation of nanohydrogels **5** (*L*-His-OCMC-g-Suc-*β*-CD carrying lapatinib and ginsenoside Rg1): The *L*-histidine modified OCMC-g-Suc-*β*-CD (100 mg) was dissolved in a PBS solution (100 mL, 10 mM, pH = 7.4). To the mixture was added anticancer drugs Lapatinib (25 mg) and Ginsenoside (25 mg), which was followed by stirring at 25°C for 24 h to form a translucent dispersion.

Nanohydrogels **6** (*L*-Arg-OCMC-g-Suc-*β*-CD carrying lapatinib and ginsenoside Rg1) were synthesized by a same procdure described above using *L*-arginine modified OCMC-g-Suc-*β*-CD as carrier.

### TEM analysis

The amino acid modified OCMC-g-Suc-β-CD (2 mg) was dispersed in deionized water (5 mL) to form a homogeneous suspension. The suspension was dropped onto a carbon-coated copper TEM grid. The grid was dried at room temperature for 5 min and treated with a phosphotungstic acid solution (10 µL). After drying using an IR lamp, the copper grid was subjected to a Hitachi-7700 transmission electron microscope to test the TEM images.

### Particle diameter and zeta potential analysis

The amino acid modified OCMC-g-Suc-*β*-CD (2 mg) was dispersed in ultrapure water (2 mL) and ultrasonicated at 25°C for 10 min. The resulting suspension was subjected to a Malvern Zetasizer Nano ZS-90 technique to test particle diameter distribution and zeta potential. The suspension was further diluted with 10 mL ultrapure water and then subjected to a dynamic light scattering instrument (DynaPro NanoStar) to determine the particle size.

### Drug loading efficiency and release behavior

The nanohydrogels **1**-**6** were centrifuged for 10 min at 3,000 r/min. The precipitate was washed with water for three times and lyophilized. The resulting solid (2 mg) was ultrasonically dissolved in methanol (5 mL) and centrifuged for 10 min at 3,000 r/min. The supernatant was diluted with methanol and subjected to a High Performance Liquid Chromatography (HPLC) machine to test the peak area. The amount of ginsenoside Rg1 was calculated according to the standard curve *y* = 18.4521*x* + 0.0021 (*R* ^2^ = 0.9990), and the amount of lapatinib was calculated using the standard curve *y* = 64.2354*x* + 0.0013 (*R* ^2^ = 0.9995). The two standard curves described above were analyzed on the HPLC machine by a published method (Chang et al., 2021). The *Y*-intercept was explained as the average peak area when the concentration of ginsenoside Rg1/lapatinib was 0, and the intercepts in the two standard curves were not statistically different from 0 (Carissimi et al., 2020). The mass of lapatinib/ginsenoside Rg1 was then calculated, and the drug loading efficiency (*DL*) was calculated using the following equation:

$$DL\left(\%\right)=\frac{W_{drug}}{W_{t}}$$

where *W*_drug_ indicates the mass of ginsenoside Rg1/lapatinib in lyophilized solid, and *W*_t_ indicates the total mass of lyophilized solid.

The drug release behavior of nanohydrogels was investigated by the HPLC method (Avgoustakis et al., 2002; Wang et al., 2023). The lyophilized solid described above (10 mg) was dispersed in a PBS buffer (10 mL, 10 mM, pH = 7.4). The suspension was dialyzed in PBS buffer (10 mM, pH = 7.4 or pH = 4.5) for 1, 2, 4, 8, 16, 24, and 48 h using a dialysis bag (3,500 MW cutoff). Then, the suspension was lyophilized, and the resulting solid was ultrasonically dissolved in methanol and centrifuged at 4,000 r/min for 10 min. The supernatant was collected and subjected to a HPLC machine to test the peak area of ginsenoside Rg1/lapatinib. The mass of ginsenoside Rg1/lapatinib after dialyzation was calculated according to the standard curves described above. The drug release rate was calculated using the following equation:

$$Drug\;release\left(\%\right)=\frac{{W_{t}\times DL\left(\%\right)-W}_{drug}}{W_{t}\times DL\left(\%\right)}$$

where *W*_drug_ indicates the mass of ginsenoside Rg1/lapatinib in lyophilized solid, and *W*_t_ indicates the total mass of lyophilized solid.

### MTT assay

Cytotoxicity of nanohydrogels **1**-**6** was analyzed through the MTT assay. In brief, human lung cancer cells (A549, ATCC) were cultured with DMEM media containing 10% FBS and 1% penicillin/streptomycin in a 5% CO_2_ incubator (Wang et al., 2020). Cells were plated on 96-well plates (10^4^ cells in each well) and incubated at 37°C for 12 h. Thereafter, cells were divided into several groups and treated with different concentrations of tested samples (0, 2, 4, 6, and 8 µg/mL). After 48 h incubation, the medium in each well was removed and replaced with 180 µL fresh medium. To each well was added 20 µL MTT solution (5 mg/mL in PBS, pH = 7.4), and all cells were cultured for another 4 h in a CO_2_ incubator. Then, the medium was removed and replaced with DMSO (150 µL), the 96-well plates were shaken for 10 min in the dark. The absorbance of each well was measured using a Microplate Reader (DYNEX, Series1SPA-0093) at 570 nm.

### Studies on anticancer activity in zebrafish

Studies on anticancer activity in zebrafish was carried out under the standard ethical guidelines of Animal Ethics Committee in Biology Institute of Shandong Academy of Sciences. The transgenic zebrafish *Tg*(*fabp10:rtTA2s-M2; TRE2:EGFP-kras* ^*V12*^) were incubated in a fish house at 28°C ± 0.5°C. The photoperiod was controlled under a 14 h/10 h light/dark cycle. Before experiments, all zebrafish were incubated overnight in a fish jar, and the female and male fish were separated by a partition board. In the next morning, the partition board was removed for mating. Three hours later, the embryos were gathered and maintained in E3 water containing 5 mM NaCl, 0.17 mM KCl, 0.33 mM CaCl_2_, 0.33 mM MgSO_4_, and 0.003% phenylthiourea.

The collected embryos were incubated in E3 water for 3 days. Then, the embryos were divided into several groups and incubated in 24-well plates. Each well contains 2 mL E3 water and 10 embryos. The Doxc group was treated with 25 μg/mL doxycycline (Doxc) for 4 days. The lapatinib group was incubated with 25 μg/mL Doxc and 4 µg/mL lapatinib for 4 days. The ginsenoside Rg1 group was incubated with 25 μg/mL Doxc and 4 µg/mL ginsenoside Rg1 for 4 days. The *L*-His-OCMC-g-Suc-*β*-CD carrying lapatinib and ginsenoside Rg1 (**5**) group was incubated with 25 μg/mL Doxc and increasing concentrations of sample **5** for 4 days (the concentration of lapatinib plus ginsenoside Rg1 was 1, 2, and 4 µg/mL). The *L*-Arg-OCMC-g-Suc-*β*-CD carrying lapatinib and ginsenoside Rg1 (**6**) group was incubated with 25 μg/mL Doxc and increasing concentrations of sample **6** for 4 days (the concentration of lapatinib plus ginsenoside Rg1 was 1, 2, and 4 µg/mL). The medium for each well was changed every day. After 4 days incubation, embryos were anesthetized with 0.08% tricaine and subjected to a fluorescence microscope (Zeiss, Jena, Germany) to get the bright and fluorescence images. The fluorescence in zebrafish generated from the *krasv* ^*12*^ oncogene labeled with EGFP (Enhanced Green Fluorescent Protein). The images were analyzed using an ImageJ software, and the date were analyzed by one-way ANOVA followed by Dunett’s test using a Graph Pad Prism 6.01 software.

## Results and discussion

### FTIR spectroscopic analysis

**FIGURE 2:**
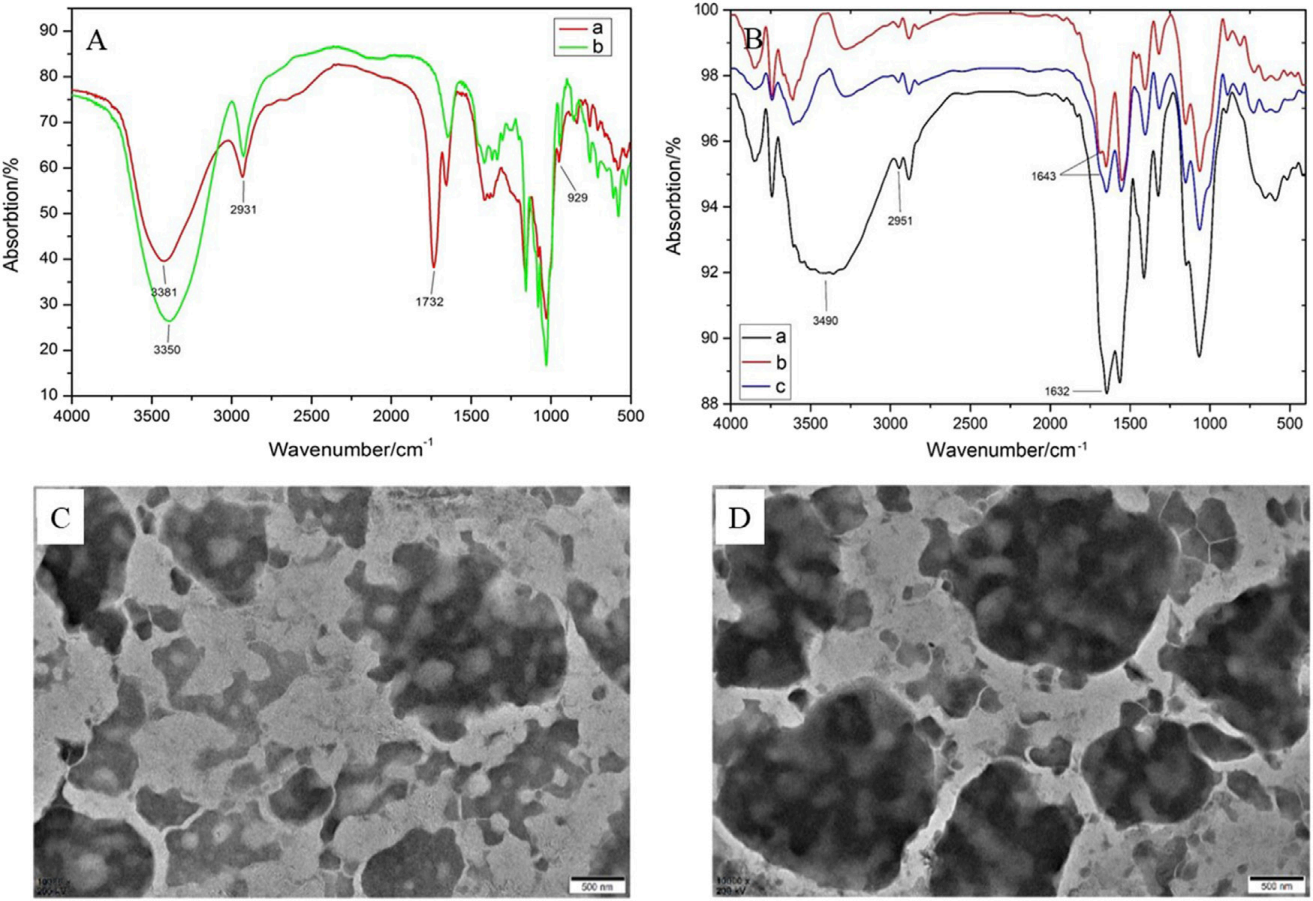
**(A)** FTIR spectra of *β*-CD and Suc-*β*-CD; **(B)** FTIR spectra of OCMC-g-Suc-*β*-CD, *L*-His-OCMC-g-Suc-*β*-CD, and *L*-Arg-OCMC-g-Suc-*β*-CD; TEM images of **(C)** *L*-His-OCMC-*g*-Suc-*β*-CD and **(D)** *L*-Arg-OCMC-g-Suc-*β*-CD.

FTIR spectra were determined by the KBr pellet method in a range of 4,000–400 cm^−1^. As depicted in Figure 2A, the characteristic peaks at 3,381 and 3,350 cm^−1^ were ascribed to the stretching vibration of–OH bond in *β*-CD and Suc-*β*-CD. The peaks at 2,931 and 941 cm^−1^ were generated by the stretching vibration of −CH bond and the skeletal vibration of the *α*-(1→4)-linkage in *β*-CD. Compared with *β*-CD, the FTIR spectrum of Suc-*β*-CD showed a new peak at 1,732 cm^−1^, which could be attributed to the stretching vibration of C=O bond in maleic acid. These results indicated that maleic anhydride had been successfully reacted with *β*-CD.

The FTIR spectra of OCMC-g-Suc-*β*-CD and amino acid modified OCMC-g-Suc-*β*-CD were shown in Figure 2B. In FTIR spectrum of OCMC-g-Suc-*β*-CD, the strong and broad peak at 3,490 cm^−1^ was ascribed to the stretching vibration of −OH bond. The peak at 2,951 cm^−1^ was generated by the stretching vibration of −CH bond. The amino groups in OCMC gave broadband at 1,000–1,200 cm^−1^, while the absorption peak at 1,632 cm^−1^ was generated to the acetylated amino group. After reacting with *L*-histidine/*L*-arginine, a new absorption peak could be observed at 1,643 cm^−1^, which was generated by the C=N group in amino acid. These results proved that *L*-histidine/*L*-arginine had been successfully grafted onto the polymer OCMC-g-Suc-*β*-CD.

### Morphology analysis

The morphology of the amino acid modified OCMC-g-Suc-*β*-CD was analyzed by TEM. As depicted in Figures 2C, D, both *L*-histidine and *L*-arginine modified OCMC-g-Suc-*β*-CD showed irregular spheroidal structure, and some pores were distributed on their surface. Various functional groups such as −OH, NH_2_, and −CO_2_H are presented in these pores, which can entrap small molecular drugs through hydrogen-bond complexation and van der Waals’ force, or ionic bonds (Chander and Kulkarni, 2021). Therefore, the pores may serve as entry points for drug molecules and also leakage the entrapped drug molecules.

### Particle diameter and zeta potential analysis

**FIGURE 3:**
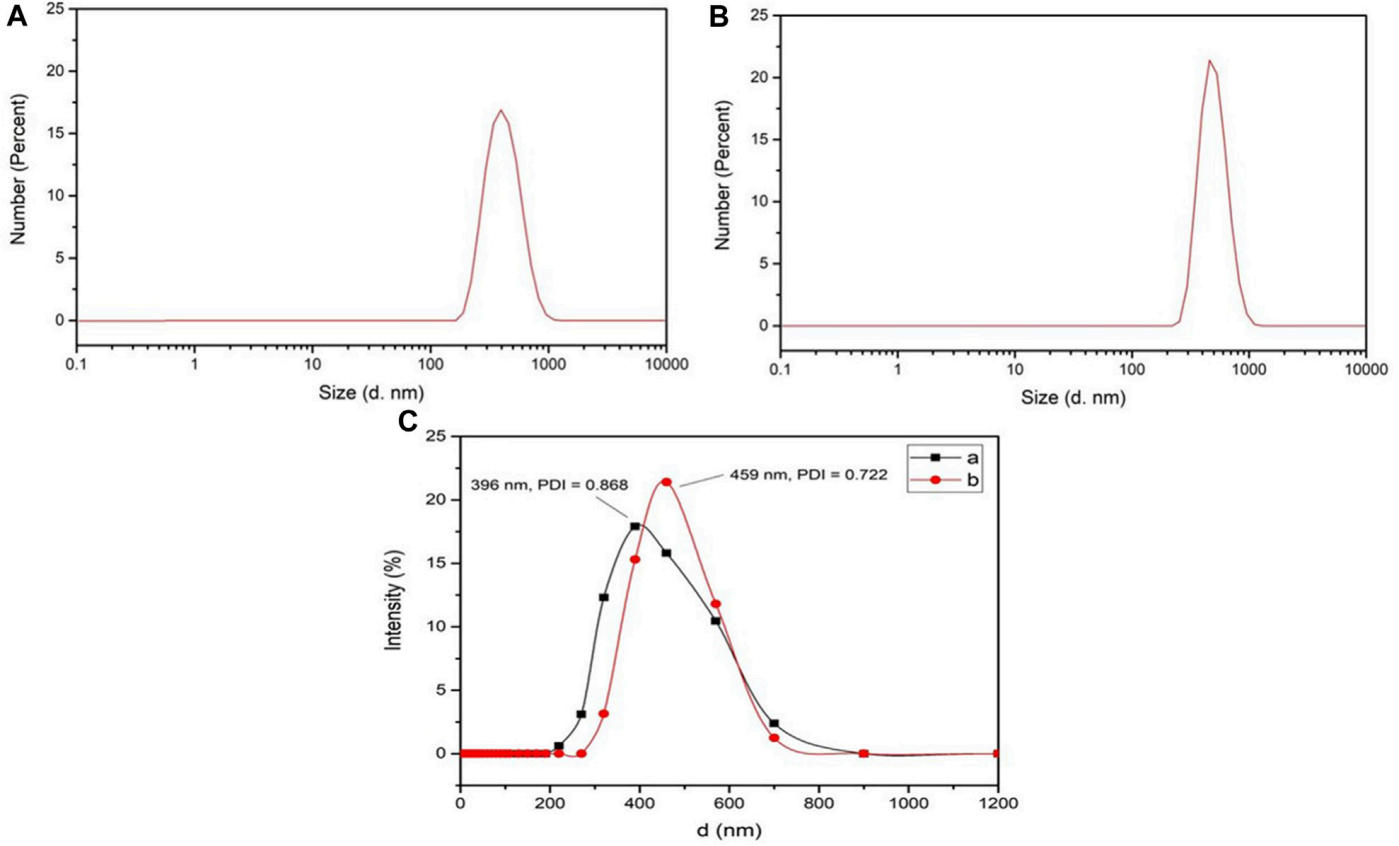
Particle size distribution of **(A)** *L*-His-OCMC-g-Suc-*β*-CD and **(B)** *L*-Arg-OCMC-g-Suc-*β*-CD; **(C)** Particle sizes and PDI of (a) *L*-His-OCMC-g-Suc-*β*-CD and (b) *L*-Arg-OCMC-g-Suc-*β*-CD.

As shown in Figures 3A, B, both *L*-histidine and *L*-arginine modified OCMC-g-Suc-*β*-CD showed a wide size distribution from 300 to 600 nm, which was small enough as anticancer drug carriers. The average particle diameters of *L*-histidine and *L*-arginine modified OCMC-g-Suc-*β*-CD were determined to be 396 ± 28.57 nm and 459 ± 19.31 nm, respectively (Figure 3C; Table 1). The polydispersity index (PDI) was 0.868 ± 0.03 and 0.722 ± 0.05, which indicated the uniformly distributed particle diameters.

**TABLE 1:** Zeta potential and particle diameters of *L*-histidine and *L*-arginine modified OCMC-g-Suc-*β*-CD nanohydrogels.

Sample	Particle diameter/nm	PDI	Zeta potential/mV
*L*-His-OCMC-g-Suc-*β*-CD	396 ± 28.57	0.868 ± 0.03	+31.06 ± 2.42
*L*-Arg-OCMC-g-Suc-*β*-CD	459 ± 19.31	0.722 ± 0.05	+31.30 ± 1.85

Zeta potential indicates the stability of nanoparticles in solution. High zeta potential (>+30 or < −30) reveals that a large number of positive or negative charges are presented on the surface of nanoparticles. The nanoparticles with the same charges (positive or negative) will be repelled with each other, thereby leading to a colloidal dispersions (Feng and Peng, 2012; Fan et al., 2013; Song et al., 2017). On the other hand, low zeta potential (<+30 or > −30) indicates that there are few electronic charges on the surface of nanoparticles, which will result in the aggregation of nanoparticles (Xie et al., 2019). In our research, we tested the zeta potential of amino acid modified OCMC-g-Suc-*β*-CD, and the results are shown in Table 1. The zeta potential of *L*-histidine and *L*-arginine modified OCMC-g-Suc-*β*-CD were tested to be +31.06 ± 2.42 mV and +31.30 ± 1.85 mV, respectively. These results indicated that there are a large number of positive charges on the surface of the synthesized polymers, which inclines to form a colloidal dispersions.

### Drug loading and release study

The standard calibration curve was arranged according to a published method (Chang et al., 2021). As shown in Table 2, the drug loading efficiency of lapatinib and ginsenoside Rg1 in *L*-histidine modified nanohydrogels **1** and **2** were calculated to be 24.05% ± 0.74% and 26.17% ± 0.32%. In contrast, *L*-arginine modified nanohydrogels **3** and **4** showed slightly low drug loading efficiency (22.48% ± 0.91% for lapatinib and 25.39% ± 0.28% for ginsenoside Rg1). The drug loading efficiency of lapatinib and ginsenoside Rg1 in nanohydrogels **5** and **6** were also determined, and the results indicated that the nanohydrogels could entrap two drugs with high drug loading efficiency (>25% for lapatinib plus ginsenoside Rg1).

**TABLE 2:** Drug loading efficiency of nanohydrogels carrying anticancer drugs lapatinib and ginsenoside Rg1.

Sample	Drug loading efficiency^a^ (%)
Nanohydrogels **1** carrying lapatinib	24.05 ± 0.74
Nanohydrogels **2** carrying ginsenoside Rg1	26.17 ± 0.32
Nanohydrogels **3** carrying lapatinib	22.48 ± 0.91
Nanohydrogels **4** carrying ginsenoside Rg1	25.39 ± 0.28
Nanohydrogels **5** carrying lapatinib and ginsenoside Rg1	13.68 ± 0.24 for lapatinib and 12.62 ± 0.13 for ginsenoside Rg1
Nanohydrogels **6** carrying lapatinib and ginsenoside Rg1	13.82 ± 0.17 for lapatinib and 14.05 ± 0.36 for ginsenoside Rg1

^a^ All measurements were conducted in triplicate and the average values were calculated.

**FIGURE 4:**
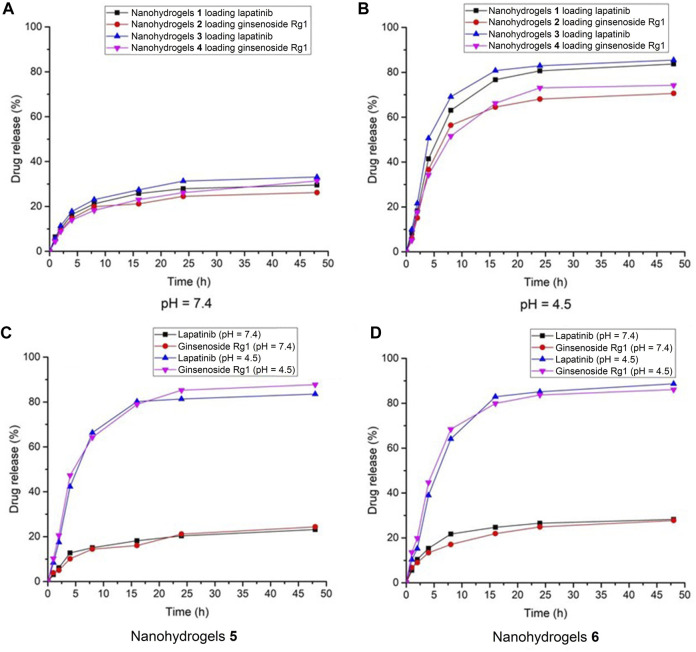
Drug release behavior of nanohydrogels **1**-**4** under **(A)** pH = 7.4 and **(B)** pH = 4.5; Drug release behavior of nanohydrogels **(C) 5** and **(D) 6**.

As shown in Figure 4A, the drug release rate of lapatinib and ginsenoside Rg1 in nanohydrogels **1**-**4** increased with increasing of dialyzation time under neutral pH condition (pH = 7.4). The drug release rates were all below 38% over 48 h dialyzation time, which revealed that the nanohydrogels showed poor release ability at neutral pH. Under acidic condition (pH = 4.5), the drug release rates were all above 70% over 48 h dialyzation time (Figure 4B). These results indicated that the nanohydrogels **1**-**4** exhibited good pH-sensitivity and could rapidly release drugs under acidic microenvironment of solid tumors (with pH ranging from 4 to 5). It should be noted that the drug release showed a burst in a short time (0–5 h) and followed by a sustained release curve. This phenomenon could be ascribed to the quick breakage of hydrogen-bond and van der Waals’ forces on the surface of nanohyrogels (Hu et al., 2016). The same drug release behavior was observed in nanohydrogels **5** and **6** carrying lapatinib and ginsenoside Rg1. In neutral pH condition (pH = 7.4), the drug release rates of lapatinib and ginsenoside Rg1 were all below 28% over 48 h dialyzation time (Figures 4C, D). But under acidic condition (pH = 4.5), the drug release rates exceeded 83%. These results indicated that the nanohydrogels could maintain lapatinib and ginsenoside Rg1 under neutral pH conditions (pH = 7.4), but could quickly release the loaded drugs under acidic condition.

### *In vitro* cytotoxicity

**FIGURE 5:**
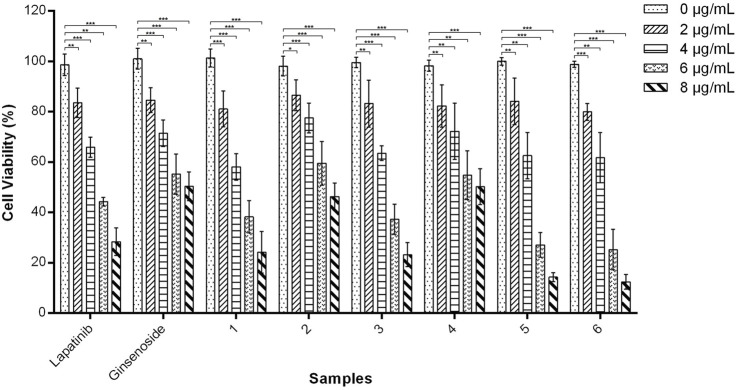
*In vitro* cytotoxicity of amino acid modified OCMC-g-Suc-*β*-CD nanohydrogels **1**-**6** carrying anticancer drugs against A549 cells after incubation for 48 h. Nanohydrogels **1**: *L*-His-OCMC-g-Suc-*β*-CD carrying Lapatinib; nanohydrogels **2**: *L*-His-OCMC-g-Suc-*β*-CD carrying ginsenoside Rg1; nanohydrogels **3**: *L*-Arg-OCMC-g-Suc-*β*-CD carrying lapatinib; nanohydrogels **4**: *L*-Arg-OCMC-g-Suc-*β*-CD carrying ginsenoside Rg1; nanohydrogels **5**: *L*-His-OCMC-g-Suc-*β*-CD carrying lapatinib and ginsenoside Rg1; nanohydrogels **6**: *L*-Arg-OCMC-g-Suc-*β*-CD carrying lapatinib and ginsenoside Rg1. The experiments were repeated 3 times, each with *n* = 3 per group. The data were presented as mean ± SEM and analyzed by one-way ANOVA followed by Dunett’s test using Graph Pad Prism 6.01 software. *p < 0.05, **p < 0.01, ***p < 0.001 VS. 0 µg/mL for each group.

Cytotoxicity of nanohydrogels **1**-**6** against A549 cells was tested using the MTT assay. As shown in Figure 5, when the concentration was below 4 µg/mL, nanohydrogels **1**-**6** exhibited moderate cytotoxicity as positive drug lapatinib. High concentration (8 µg/mL) of nanohydrogels resulted in low cell viability (below 40%). Nanohydrogels loaded with lapatinib and ginsenoside Rg1 (**5** and **6**) exhibited higher cytotoxicity than nanohydrogels **1**-**4**. These results indicated that the coexistence of lapatinib and ginsenoside Rg1 may induced synergistic action of the two drugs. Ginsenoside Rg1 showed poor cytotoxicity against A549 cells, but could significantly improve the cytotoxicity of lapatinib.

### Anticancer investigation in a zebrafish model

**FIGURE 6:**
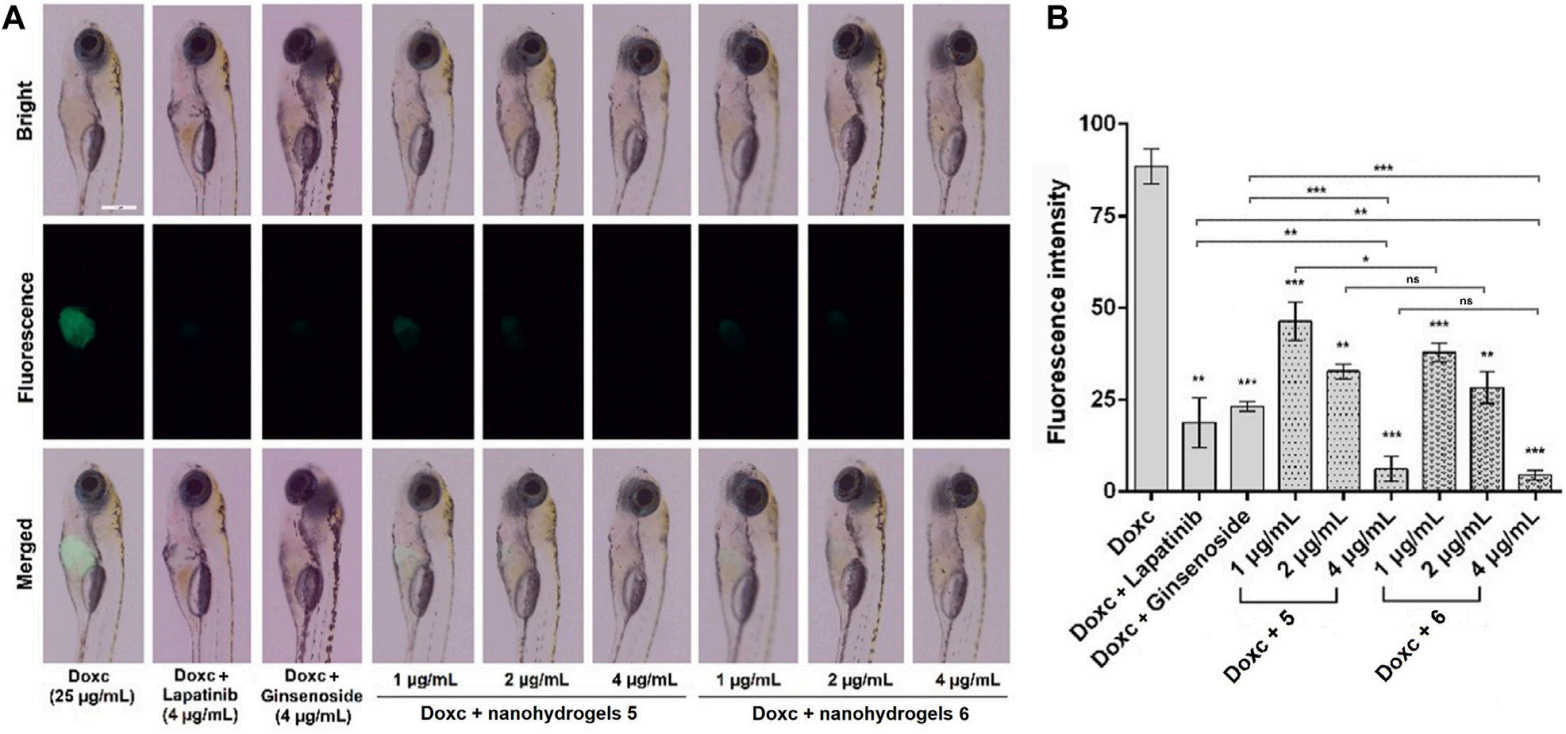
Images of zebrafish **(A)** and fluorescence intensity in zebrafish liver **(B)** exposed to Doxc (25 μg/mL) and tested compounds including lapatinib (4 µg/mL), ginsenoside Rg1 (4 µg/mL), nanohydrogels **5** (the concentration of lapatinib plus ginsenoside Rg1 1, 2, and 4 µg/mL), and nanohydrogels **6** (the concentration of lapatinib plus ginsenoside Rg1 was 1, 2, and 4 µg/mL). Scale bar is 500 μm. Data were analyzed by one-way ANOVA followed by Dunett’s test using a Graph Pad Prism 6.01 software. The results were expressed as mean ± SEM, **p < 0.01, ***p < 0.001 VS. Doxc.

Anticancer investigation of nanohydrogels **5** and **6** was carried out in a transgenic zebrafish *Tg*(*fabp10:rtTA2s-M2; TRE2:EGFP-kras* ^*V12*^) model. The transgenic zebrafish reported by Gong group has been widely used as a model for anticancer drug screening (Nguyen et al., 2012; Yan et al., 2017; Zhu et al., 2021; Yu et al., 2022). In the presence of Doxc (25 μg/mL), the liver area of zebrafish can express *EGFP-kras* ^*v12*^ oncogene. Then, we can observe a green fluorescence in zebrafish liver generated by the EGFP (Enhanced Green Fluorescent Protein) (Nguyen et al., 2012). As shown in Figure 6A, zebrafish exposed to Doxc (25 μg/mL) for 4 days showed strong green fluorescence, which indicated the expression of *EGFP-kras* ^*v12*^ oncogene in zebrafish liver. After co-treatment of zebrafish with Doxc (25 μg/mL) and lapatinib (4 µg/mL)/ginsenoside Rg1 (4 µg/mL) for 4 days, the liver of zebrafish showed weak fluorescence and the fluorescence intensity was significantly decreased (Figure 6B). This information indicated that lapatinib or ginsenoside Rg1 inhibited the expression of *EGFP-kras* ^*v12*^ oncogene and showed moderate anticancer activity in zebrafish. In contrast, the fluorescence in zebrafish liver was disappeared in the presence of nanohydrogels **5** and **6** (4 µg/mL), which indicated the absolutely inhibition of *EGFP-kras* ^*v12*^ oncogene and the high anticancer activity of the synthesized nanohydrogels. The *L*-arginine modified nanohydrogels loaded with lapatinib and ginsenoside Rg1 (**6**) showed higher anticancer activity than nanohydrogels **5**. The zebrafish model can also be used for investigating the toxicity of tested compounds, and high toxicity compounds will induce high mortality in zebrafish larvae. In our experiments, zebrafish larvae treated with different concentrations of tested samples (1, 2, and 4 µg/mL) showed no obvious increase in mortality, which indicated the low toxicity of nanohydrogels.

## Conclusion

In this paper, two novel amino acid modified OCMC-g-Suc-*β*-CD polymers were prepared. Morphology analysis indicated that the polymers had irregular spheroidal structure with some pores distributed on their surface, which was suitable for entrapping anticancer through hydrogen-bond complexation and van der Waals’ force. The polymers showed small average particle diameters (396 ± 28.57 nm and 459 ± 19.31 nm) with appropriate PDI (0.868 ± 0.03 and 0.722 ± 0.05), and the zeta potential were above +30 mV. The polymers were successfully used for preparing nanohydrogels loaded with anticancer drugs lapatinib and ginsenoside Rg1 with high drug loading efficiency. More importantly, the nanohydrogels showed low drug release efficiency under neutral pH conditions (pH = 7.4), but with rapid drug release rates under the acidic microenvironment of solid tumors (pH = 4–5). *In vitro* and *in vivo* anticancer investigation indicated that the *L*-arginine modified OCMC-g-Suc-*β*-CD nanohydrogels loaded with lapatinib and ginsenoside Rg1 (**6**) could significantly inhibit the proliferation of A549 cells and the overexpression of *EGFP-kras* ^*v12*^ oncogene in zebrafish. All these results indicated that nanohydrogels **6** should be potential candidate for further biological investigations.

## Data Availability

The original contributions presented in the study are included in the article/Supplementary Material, further inquiries can be directed to the corresponding authors.

## Abbreviations

OCMC, Carboxymethylated chitosan; *β*-CD, *β*-Cyclodextrin; FTIR, Fourier transform infrared; TEM, Transmission electron microscope; DLS, Dynamic light scattering; CS, Chitosan.
